# Supplementary material for: Recombinant Ixodes scapularis Calreticulin Binds Complement Proteins but Does Not Protect Borrelia burgdorferi from Complement Killing
Source: Pathogens. 2024 Jul 3;13(7):560. doi: 10.3390/pathogens13070560 (PMC11280304; doi:10.3390/pathogens13070560)
Supplement: Supplementary file 1 [file pathogens-13-00560-s001.zip › Supplementary Figure S1- Recalcification time assay.pdf]

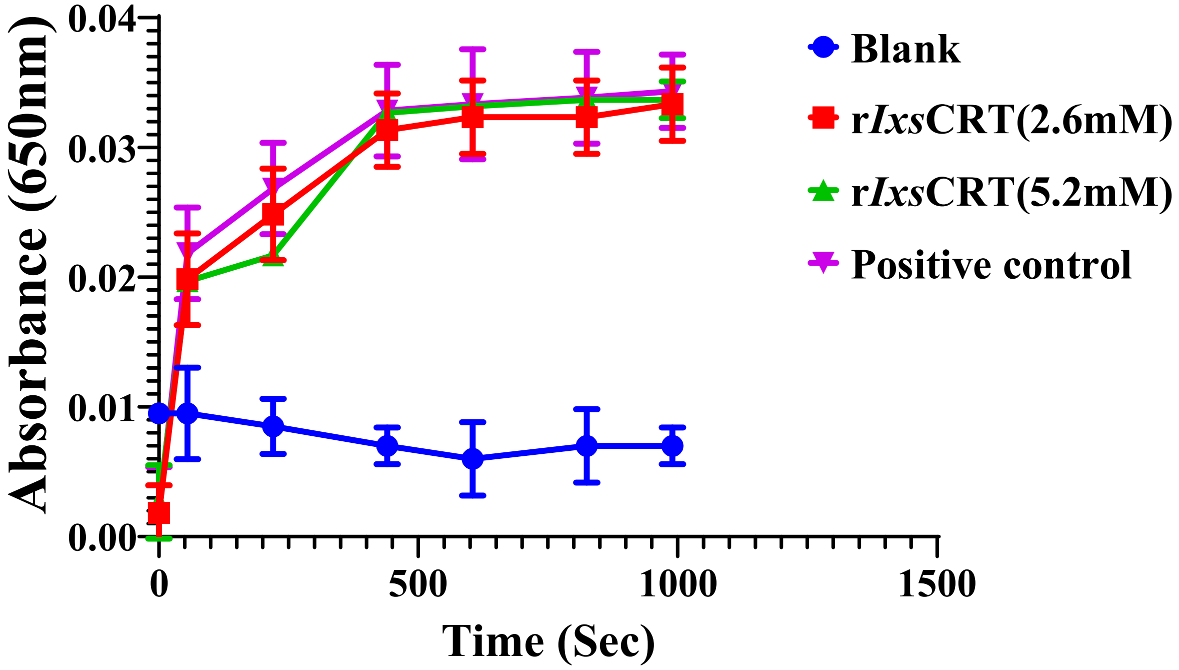

Figure S1: Recalcification time assay demonstrates that r*Ixs*CRT does not delay plasma clotting. To investigate the impact of r*Ixs*CRT on plasma clotting time via the common pathway, a recalcification time assay was conducted. Universal coagulation reference human plasma (15 µl) was incubated with r*Ixs*CRT at 2.6mM (red) and 5.2mM (green) for 15 minutes at 37°C. Subsequently, 150 mM $CaCl_2$ was added, and clotting (recalcification) time (λ650nm) was recorded every 20 seconds for 20 minutes. No significant delay in plasma clotting time was observed to the plasma incubated with r*Ixs*CRT or without r*Ixs*CRT (pink), indicating that r*Ixs*CRT does not affect blood clotting via the recalcification time pathway. Plasma with buffer and without $CaCl_2$ served as the blank (blue). The assay was performed twice in triplicate, and statistical analysis was conducted using Student's t-test in GraphPad Prism 9.
